# Transgenic systems for unequivocal identification of cardiac myocyte nuclei and analysis of cardiomyocyte cell cycle status

**DOI:** 10.1007/s00395-015-0489-2

**Published:** 2015-04-30

**Authors:** Alexandra Raulf, Hannes Horder, Laura Tarnawski, Caroline Geisen, Annika Ottersbach, Wilhelm Röll, Stefan Jovinge, Bernd K. Fleischmann, Michael Hesse

**Affiliations:** Institute of Physiology I, Life and Brain Center, University of Bonn, Sigmund-Freud-Strasse 25, 53105 Bonn, Germany; Lund Strategic Research Center for Stem Cell Biology and Cell Therapy, Lund University, Lund, Sweden; DeVos Cardiovascular Research Program, Van Andel Institute/Spectrum Health, Grand Rapids, USA; Department of Cardiac Surgery, University of Bonn, Bonn, Germany; Pharma Center Bonn, Bonn, Germany

**Keywords:** Cardiac myocyte, Cell cycle, Genetically altered mice, Proliferation, miRNA

## Abstract

Even though the mammalian heart has been investigated for many years, there are still uncertainties in the fields of cardiac cell biology and regeneration with regard to exact fractions of cardiomyocytes (CMs) at different developmental stages, their plasticity after cardiac lesion and also their basal turnover rate. A main shortcoming is the accurate identification of CM and the demonstration of CM division. Therefore, an in vivo model taking advantage of a live reporter-based identification of CM nuclei and their cell cycle status is needed. In this technical report, we describe the generation and characterization of embryonic stem cells and transgenic mice expressing a fusion protein of human histone 2B and the red fluorescence protein mCherry under control of the CM specific αMHC promoter. This fluorescence label allows unequivocal identification and quantitation of CM nuclei and nuclearity in isolated cells and native tissue slices. In ventricles of adults, we determined a fraction of <20 % CMs and binucleation of 77–90 %, while in atria a CM fraction of 30 % and a binucleation index of 14 % were found. We combined this transgenic system with the CAG-eGFP-anillin transgene, which identifies cell division and established a novel screening assay for cell cycle-modifying substances in isolated, postnatal CMs. Our transgenic live reporter-based system enables reliable identification of CM nuclei and determination of CM fractions and nuclearity in heart tissue. In combination with CAG-eGFP-anillin-mice, the cell cycle status of CMs can be monitored in detail enabling screening for proliferation-inducing substances in vitro and in vivo.

**Electronic supplementary material:**

The online version of this article (doi:10.1007/s00395-015-0489-2) contains supplementary material, which is available to authorized users.

## Introduction

The mammalian heart is composed of different cell types such as endothelial cells, fibroblasts, smooth muscle cells, and cardiomyocytes (CMs). The latter provide the contractile properties of the heart muscle and CM loss in pathologies, such as myocardial infarction or cardiomyopathy, leads to functional impairment and eventually heart failure [16]. While there is mutual consent that CMs make up the majority of the heart mass [20], there is a discrepancy regarding the fraction of CMs in the mammalian heart which is estimated to be composed of 15–35 % CMs [25, 37, 42], as addressed by histology but not specific cell markers [3]. A recent report, using flow cytometry, claims that mouse hearts exceptionally consist of 55 % of CMs [2]. Also identifying CM nuclei is challenging in cardiac sections in which CM cytoplasm is identified either by immunohistological staining for structural proteins or by transgenic CM-specific overexpression of fluorescence proteins. In those sections, non-CM nuclei in close proximity to CM cytoplasm cannot be distinguished from CM nuclei leading to false positives [1].

The majority of CMs are polyploid, caused either by an increase in nuclearity with ~90 % binuclear ventricular CMs in mouse [37, 43] and rat [21], and 25.5–61.9 % in humans [8, 29] or by increased nuclear ploidy up to 8N in humans [24] and 16N in mice [43]. Polyploid cells are thought to be unable to undergo cell division, but are still able to enter the cell cycle and to perform DNA synthesis resulting in CMs with further increased ploidy [14, 23]. Heart regeneration is commonly analyzed with proliferation markers localized in the nucleus, such as PCNA, Ki-67, or pHH3 or Thymidine analogs such as BrdU or ^3^H-Thymidine. The results of these studies depend strongly on the unequivocal identification of CM nuclei, which is error-prone without the use of a specific nuclear CM marker [1]. This could be one of the major reasons for the discrepancies concerning the estimates of annual turnover rates of adult mouse CMs, ranging from 0.76 % [33] up to 80 % [17] and the regeneration potential after experimental lesions, estimated from 0 % [15, 44] up to 15.3 % [18]. Using a new in vivo proliferation marker, the eGFP-anillin system, we recently showed that adult CMs undergo endoreduplication (DNA replication without karyokinesis or cytokinesis) rather than cell division after cardiac lesion in mice [15].

Two challenges arise from the above-mentioned discrepancies, firstly the unambiguous identification of CM nuclei and secondly the confirmation of CM division and not mere cell cycle activity.

Here, we report the generation of a reporter system that directly visualizes CM nuclei in vitro and in vivo, by fusing the red fluorescence protein mCherry (mCh) to human histone 2B (H2B) and expressing it in CMs by use of the αMHC (Myh6) promoter. We demonstrate that this fusion protein accurately labels CM nuclei and allows the determination of CM portions and nuclearity during postnatal development of the mouse heart and after cardiac injury. We further combine αMHC-H2B-mCh mice with the CAG-eGFP-anillin mouse line for unambiguous visualization of cell cycle progression and division of postnatal CMs and for establishing a screening assay for cell cycle-modifying substances.

## Methods

### Generation of the αMHC-hH2BmCh-IRES-Puro^R^ vector

The pIRES2-eGFP vector (CLONTECH Laboratories, Inc.) was digested with AseI and NheI, blunt-ended and religated. Thereby the CMV IE promoter was excised. EGFP was replaced by the Puromycin^R^ (PuroR) cassette (HindIII-ClaI fragment) of the Cre-Pac vector. The resulting vector (pIRES2-Puro^R^) was opened with BglII, blunt-ended and subsequently digested with XhoI. The mouse αMHC promoter [5.5 kb BamHI(blunt)-SalI fragment] of the αMHC-pBK plasmid (kindly provided by J. Robbins, University of Cincinnati, Cincinnati, OH) was inserted into the opened pIRES2-Puro^R^-vector (pαMHC-IRES2-Puro^R^).

The pCAG-hH2B-mCh-IRES-Puro^R^ expression vector was a gift from Heiko Lickert, Helmholtz Zentrum München. The vector was digested with NotI, blunt-ended, and cut with AccIII. The excised H2BmCh-IRES-Puro fragment was ligated into the EcoICRI-AccIII opened pαMHC-IRES-Puro^R^ vector, thereby replacing the IRES2-sequence and part of the Puro^R^ cDNA of the vector. The resulting vector αMHC-hH2B-mCh-IRES-Puro^R^ (αMHC-H2B-mCh) was verified by restriction analysis and sequencing.

### Generation and cultivation of transgenic ESC clones

G4 hybrid ESCs [12] were cultured in Knockout-Dulbecco’s modified Eagle’s medium (DMEM), high-glucose, supplemented with 15 % v/v fetal calf serum (FCS), 0.1 mM nonessential amino acids, 2 mg/ml l-Glutamine, 50 μg/ml each penicillin and streptomycin, 0.1 mM β-mercaptoethanol, and 500 U/ml Leucemia inhibitory factor (LIF). The ESCs were kept on irradiated neomycin-resistant mouse embryo fibroblasts (Millipore).

For generation of transgenic ESCs 5 × 10^6^ cells were mixed with 30 µg of linearized plasmid DNA in PBS and electroporated at 250 V and 500 µF, 1 pulse, using a Bio-Rad Gene Pulser. The cells were plated on two 100-mm plates. Selection for neomycin-resistant cells started 2 days after electroporation by adding 165 µg/ml G418 to the medium. Resistant colonies were picked onto mouse embryonic fibroblast-coated 24-well plates, propagated and analyzed for mCh expression.

### Differentiation of transgenic ESCs and purification of CMs

ESC differentiation was performed using mass culture. In brief, 2.0 × 10^6^ ESCs were suspended in 10 ml differentiation medium (Iscove’s Modified Dulbecco’s Medium (IMDM), supplemented with 20 % FCS, 0.1 mM nonessential amino acids, 50 μg/ml each penicillin and streptomycin, 0.1 mM β-mercaptoethanol in the absence of LIF and cultivated on a horizontal shaker at 70–80 rpm, 37 °C, and 5 % CO_2_ for 2 days. Half of the EBs were further cultivated in a CELLSPIN 500 (INTEGRA biosciences) spinner flask. To select for CMs, puromycin treatment (0.5–1.5 µg/ml depending on the resistance of particular clone) was started on day 10 of differentiation in suspension culture. For isolation of CMs on d11 and d13, the EBs were dissociated with 0.125 % Trypsin/EDTA for 5 min. Treatment was stopped with differentiation medium and the cells were centrifuged (210 g, 5 min). ~10^5^ cells were plated on coverslips, pre-treated with 10 µg/ml fibronectin in 24-well tissue plates, cultured for 48 h in differentiation medium, and for further purification of CMs puromycin was added. Cells were fixed with 4 % paraformaldehyde (PFA) at room temperature (RT) for 30 min.

### Copy number determination by qPCR

The copy number of the H2B-mCh transgene was determined in different ESC clones applying a TaqMan-based qPCR assay as described by the manufacturer (Applied Biosystems). For the transgene detection, a Carboxyfluorescein (FAM)-coupled mCh probe (Applied Biosytems) was used and as genomic reference a VIC-coupled probe for the Transferrin receptor was chosen. Both probes were analyzed in triplicates for each gDNA (200 ng). QPCR was performed in a Rotor-Gene 6000 (Corbett).

### Transgenic αMHC-H2BmCh-IRES-Puro^R^ mice and myocardial infarction

All animal experiments were performed in accordance with the guidelines from Directive 2010/63/EU of the European Parliament on the protection of animals used for scientific purposes and were approved by the Animal Care Committees (8.87-50.10.31.08.199 and 8.87-50.10.37.09.238). Transgenic ESC clones were screened for a proper karyotype (40 chromosomes) and aggregated with diploid morula stage CD1 embryos as described previously [26]. Ventricular cryolesions were generated in 10-week-old mice with 3.5-mm liquid-nitrogen-cooled copper probes as described earlier [31]. Briefly, mice were anesthetized with 4 Vol% Isoflurane, 0.6 l/min O_2_, 0.4 l/min N_2_O, intubated and ventilated. The concentration of Isoflurane was reduced to 1–2 Vol%. The heart was exposed by thoracotomy. For local anesthesia, 0.1 ml of a 0.5 % Bupivacaine solution was injected into the intercostal musculature. Cryolesions at the anterolateral left ventricular wall were generated using liquid-nitrogen-cooled copper probes 3.5 mm in diameter. For ligation the left anterior descending (LAD) coronary artery was occluded directly distal of the left atrial auricle. For peri-operative analgesia, 20 mg/kg s.c Metamizol was used and 5 mg/kg s.c. Carprofen was used for analgesia post operation for 5 days. Neonatal mice were euthanized by decapitation and adult mice by cervical dislocation.

### Left ventricular catheterization and hemodynamics

Left ventricular catheterization was performed on 15-week-old H2B-mCh and CD1 wt mice by a blinded investigator (for detail see [11]). Briefly, a 1.4 F Millar Aria1 catheter (Millar Instruments Inc., Houston, USA) was inserted under general anesthesia via the right carotid artery into the left ventricle, and pressure-volume (PV)-loops were recorded online. For analysis, Millar PVAN software was used. After the experiment, mice were sacrificed and hearts were harvested for histological analysis.

### Dissociation of postnatal mouse hearts and miRNA transfection

Mice were sacrificed on d2/d3/d7 after birth; the hearts were prepared and analyzed for H2B-mCh expression by using a macroscope (AxioZoom.V16, Zeiss). Atria and ventricles of the transgenic hearts were separated and dissociated using the Neonatal heart dissociation kit (Miltenyi Biotec) without using the gentle MACS Dissociator. The heart tissue was dissected by slowly pipetting every 15 min. 7500 cells were seeded per well on a 0.001 % fibronectin-coated 384-well µ-clear microtiter plate (Greiner). Cells were incubated in differentiation medium (see above) at 37 °C and 5 % CO_2_. For analysis of the binucleation index, cells were fixated after overnight adhesion to the dish.

In order to transfect postnatal CMs, ventricles of αMHC-H2B-mCh/CAG-eGFP-anillin double transgenic hearts were dissociated as described above. 5 µl of 500 nM Stock solutions of hsa-miR-199a-3p or miR mimic, Negative control #1 (Ambion, Life Technologies) were incubated with 0.2 µl Lipofectamine RNAi Max (Invitrogen) in 14.8 µl OPTI-MEM on 0.001 % fibronectin-coated 384-well µ-clear microtiter plates (Greiner) for 20 min at RT. Afterward, 7500 dissociated cells were added in a volume of 60 µl differentiation medium. Medium was changed after 48 h and the cells were further incubated for 24 h.

### Harvesting of mouse hearts

Heparinized adult mice were sacrificed by cervical dislocation and the hearts were harvested. After cannulation of the ascending aorta, the hearts were perfused with PBS, followed by perfusion with 4 % PFA, and further fixation overnight. Embryonic and postnatal hearts were excised and immersion fixated in 4 % PFA. Hearts were dehydrated in 20 % sucrose solution and mounted in O.C.T. compound.

### Langendorff dissociation

Ventricular myocytes from adult mice were enzymatically isolated by Langendorff perfusion as previously described [39], with minor variations. Briefly, animals were killed by cervical dislocation. The heart was dissected and perfused using oxygenated Ca^2+^-free Tyrode solution (135 mM NaCl, 4 mM KCl, 1 mM MgCl_2_, 2.5 mM HEPES, 5 mM Glucose, pH 7.4) with 25 mM 2,3-Butanedione monoxime (BDM) for 5 min. The enzymatic digestion with 1 mg/ml (activity 0.199 U/mg) Collagenase B and 0.033 mg/ml Trypsin was performed in oxygenated Tyrode solution with 25 mM BDM and 50 µM CaCl_2_ for 8–12 min and after manually dissecting the tissue the reaction was stopped in stopping solution (oxygenated Tyrode solution with 25 mM BDM, 50 µM CaCl_2_ and 5 % FCS). The cell suspension was filtered through a 100-µm Cell strainer and centrifuged (700 rpm, 1 min). Isolated cells were plated on 8 µg/ml laminin-coated coverslips in 24-well plates in differentiation medium (see above) at 37 °C, 5 % CO_2_ for 5 h and fixated with 4 % PFA solution afterward.

### Histology and immunofluorescence stainings

Following PFA fixation, all tissues and EBs were incubated in 20 % sucrose in PBS prior to cryopreservation in Tissue Tek O.C.T. compound (Sakura Finetek Europe B.V.). Sectioning of 10 µm/50 µm cryoslices was performed with a cryotome CM 3050S (Leica).

Fixated cells and tissue slices were stained for the following differentiation and proliferation markers (in 0.2 % Triton X in PBS, supplemented with 5 % donkey serum; 2 h at RT): α-actinin (1:400, Sigma–Aldrich A7811), cTropT (1:50, DSHB Hybridoma Product RV-C2), PCM-1 (1:200, Cell Signaling G2000), and Aurora B kinase (1:400, Sigma-Aldrich A5102). For the PCM-1 staining a short antigen-retrieval with 10 mM sodium citrate, pH 6.0, was performed in a KOS Microwave (Milestone, program: 4 min 70 °C, 3 min 85 °C, 4 min 90 °C, 5 min 93 °C), which allowed conservation of the H2B-mCh fluorescence. Primary antibodies were visualized by secondary antibodies conjugated to Cy5 (1:400, Jackson ImmunoResearch) diluted in 1 µg/ml Hoechst 33342 (nuclei staining) at RT for 1 h. Fluorescein Griffonia Simplificiosa Lectine (GSL, Vector Laboratories) was diluted 1:100 and stained at RT for 1 h. Immunostainings were documented with an inverted fluorescence microscope (Axiovert 200; Carl Zeiss MicroImaging, Inc.) equipped with a slider module (ApoTome; Carl Zeiss MicroImaging, Inc.). Sirius red, hematoxylin, and eosin stainings were performed using standard histology protocols. Pictures were acquired with an AxioStar Plus microscope (Zeiss).

For quantification of CM nuclei fractions in different regions of the heart, 10-µm thick transversal cryo-sections were analyzed (zone 1: section at atria level, zone 2: section through the middle of the ventricle, determined by length measurement of a macroscopic picture of each heart, zone 3: section in ~200 µm distance from the apex). The following 25× pictures of cross-sectioned CMs were analyzed per mouse (*n* = 3 per time point) and time points (P3, P7, 9 W): zone 1: left *A* atrium, *LV* left ventricle compact zone, LV trabecular zone, *RV* right ventricle compact zone, RV trabecular zone, zone 2: LV compact zone, LV trabecular zone, RV compact zone, RV trabecular zone, layer 3: LV compact zone, LV trabecular zone.

### Determination of binuclearity and fractions of CM nuclei in thick slices

PFA-fixated, cryopreserved hearts were sliced into 50-µm thick sections with a cryotome CM 3050S (Leica) and treated with RNAse A (20 µg/ml) in wash buffer (0.5 M NaCl, 0.1 M Tris pH 7.5, 50 mM EDTA) for 1 h at 37 °C. Slices were incubated with 1 µM TO-PRO3 iodide (642/661) (Molecular Probes) and Fluorescein wheat germ agglutinine (WGA) (1:100, Vector Laboratories) at 4 °C overnight. Image stacks were acquired with an inverted confocal laser scanning microscope (Nikon Eclipse Ti) equipped with a ×40/1.15 NA water-dipping objective (ApoLWD 40× WI SDIC N2). Stacks with a z-step width of 0.5 µm were recorded. Excitation wavelengths for WGA, H2B-mCh, and TO-PRO 3 were 488, 543, and 642 nm, respectively. Binucleation was determined in z-stacks manually by scrolling through the different layers of the stack. Only cells that lay completely within the z-stack (WGA staining visible in every dimension) were quantified.

The number of CM nuclei (H2B-mCh^+^) and all nuclei (TO-PRO3^+^) was determined in 3D-reconstructions using the automatic 3D analysis module of NIS Elements. The result was verified by manually correcting for doublets, which were nuclei in very close proximity to each other, that were not correctly separated by the software. Virtual longitudinal and cross sections (0.5 µm) were generated using NIS Elements. Per thick slice ≥3 longitudinal sections with a distance of 10 µm to each other were manually analyzed for H2B-mCh signals and TO-PRO3 signals. In virtual cross sections, the distance between the analyzed sections (*n* ≥ 4 per thick slice) was 40 µm.

### Nuclei isolation and flow cytometry

Nuclei isolation experiments were performed as reported earlier [5]. In brief, frozen adult H2B-mCh hearts were cut into pieces manually and put in ice cold lysis buffer (in mM: 350 Sucrose; 5 CaCl_2_; 5 C_4_H_6_MgO_4_–4H_2_O; 2.0 EDTA; 0.5 EGTA; 10 Tris–HCl (pH 8.0); 1 DTT). The heart pieces were homogenized with a T-25 Ultra-Turrax^®^ probe homogenizer (IKA) at 24 k rpm for 10 s and then using a glass dounce to further homogenize the tissue and to free the nuclei. The crude lysate was passed through a 100- and 70-μm nylon mesh cell strainer (BD Biosciences), consecutively, and then centrifuged at 700 g for 10 min in (4 °C). The nuclei pellet was re-suspended in sucrose buffer containing (in mM) the following: 2100 sucrose; 5 C_4_H_6_MgO_4_–4H_2_O; 1 DTT; 10 Tris–HCl (pH 8.0). By using gradient centrifugation, the sample was centrifuged at 13,000 g for 60 min in 4 °C. The nuclei pellet was re-suspended in nuclei storage buffer containing (in mM) the following: 440 sucrose; 70 KCl; 10 MgCl_2_; 10 Tris–HCl (pH 7.2); 1.5 spermine. Nuclei were stained with rabbit anti-mouse PCM-1 (Cell Signaling Tech.) overnight in 4 °C. Primary antibody was visualized with secondary antibody conjugated to FITC (Jackson ImmunoResearch) and DNA was stained with DAPI. Cytometric acquisition was performed on Aria III (BD Biosciences) using the 70-µm nozzle. Positive gates were set according to unstained controls, FMO controls, and single staining controls. Analysis was done using FlowJo™ Version V10.

### Statistical analysis

Data are depicted as mean ± SEM. Statistical significance was determined by Student’s unpaired* t* test or 1way ANOVA with Bonferroni’s multiple comparison test. *p* < 0.05 was considered statistically significant.

## Results

### Characterization of αMHC-hH2B-mCh-IRES-Puro^R^ transgenic ESC clones

**Fig. 1:**
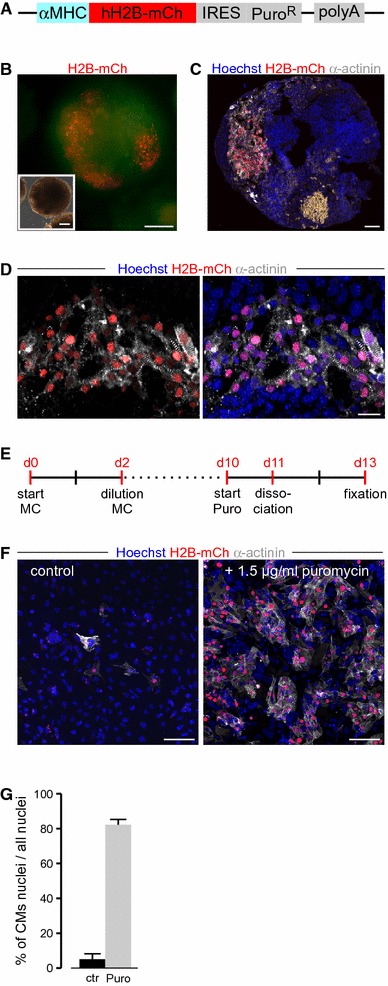
Identification of CM nuclei in vitro. **a** Expression construct used for the generation of αMHC-H2B-mCh transgenic mouse ESCs. **b** H2B-mCh expression in beating areas of EBs at d14 of differentiation. *Scale bar* 200 µm. **c** Section of an H2B-mCh^+^ EB indicates specific expression of the fusion protein in CMs, identified by α-actinin staining. *Scale bar* 50 µm. **d** Close-up of a CM-rich region of an H2B-mCh^+^ EB. CMs were identified by α-actinin staining. *Scale bar* 20 µm. **e** Protocol for differentiation of ESCs and selection for CMs. **f** Selection for CMs in H2B-mCh transgenic EBs by puromycin treatment. Left picture shows dissociated EBs without selection and right picture with puromycin treatment for 3 days. H2B-mCh^+^ CMs were verified by α-actinin staining. *Scale bar* 100 µm. **g** Quantification of the selection for CMs in αMHC-H2B-mCh transgenic EBs (d13) treated with puromycin for 3 days (*n* = 3)

To establish a system that allows unequivocal identification of CM nuclei in vitro and in vivo, we cloned a fusion cDNA consisting of human histone 2B (H2B) and mCh behind the cardiac-specific α-Myosin-heavy-chain (αMHC, Myh6) promoter (Fig. 1a). The construct also contained an internal ribosomal entry site (IRES) followed by a Puromycin resistance cassette for the enrichment of CMs. We used a fusion protein with H2B for two main reasons: First, for determination of the cell cycle activity in CMs nuclear markers such as BrdU or Ki-67 are used most commonly, rendering the clear identification of CM nuclei of crucial importance [1]. Second, after dissolving of the nuclear membrane in M-phase, transgenic systems using a nuclear localization signal encounter a translocation of the fluorescence protein to the cytoplasm, which is hard to detect for cell tracking image analysis algorithms [27]. Additionally, H2B fusion proteins have a very long half-life [40], therefore maintaining the nuclear signal during potentially fluctuating activity of the αMHC promoter.

For generation of transgenic mice and in vitro analysis of H2B-mCh expression in CMs, we generated stably transfected mouse embryonic stem cell (mESC) lines and differentiated these into embryonic bodies (EBs). The expression of the reporter construct became visible around day 8 of differentiation (day 8). This correlated with the appearance of the first spontaneous beating cell clusters in the EBs and H2B-mCh expression was restricted to these areas (Fig. 1b, Suppl. video 1). Cryoslices of fixated EBs at day 10 revealed an exclusive expression of the H2B-mCh protein in the nuclei of α-actinin^+^ CMs (Fig. 1c, d). On day 10 puromycin treatment was started for 3 days to enrich for ESC-derived CMs. During puromycin treatment, EBs were dissociated and the cells replated (Fig. 1e), followed by quantification of H2B-mCh^+^/α-actinin^+^ CMs (Fig. 1f), which revealed a ~16-fold enrichment of CMs (Fig. 1g). The overlap of H2B-mCh expression and α-actinin staining was 96 ± 1.5 %, revealing high specificity of the transgene. In summary, the αMHC-H2B-mCh system can identify CM nuclei in vitro and CMs derived from the transgenic ESCs are viable and can be enriched by puromycin treatment.

### H2B-mCh specifically marks CM nuclei in transgenic hearts

From a characterized αMHC-H2B-mCh ESC clone with only one transgene integration, we generated a transgenic mouse line by aggregation with diploid embryos from wildtype CD1 mice. The chimeric mice provided germ-line transmission and the transgenic progeny was viable, fertile, and had a normal lifespan.

**Fig. 2:**
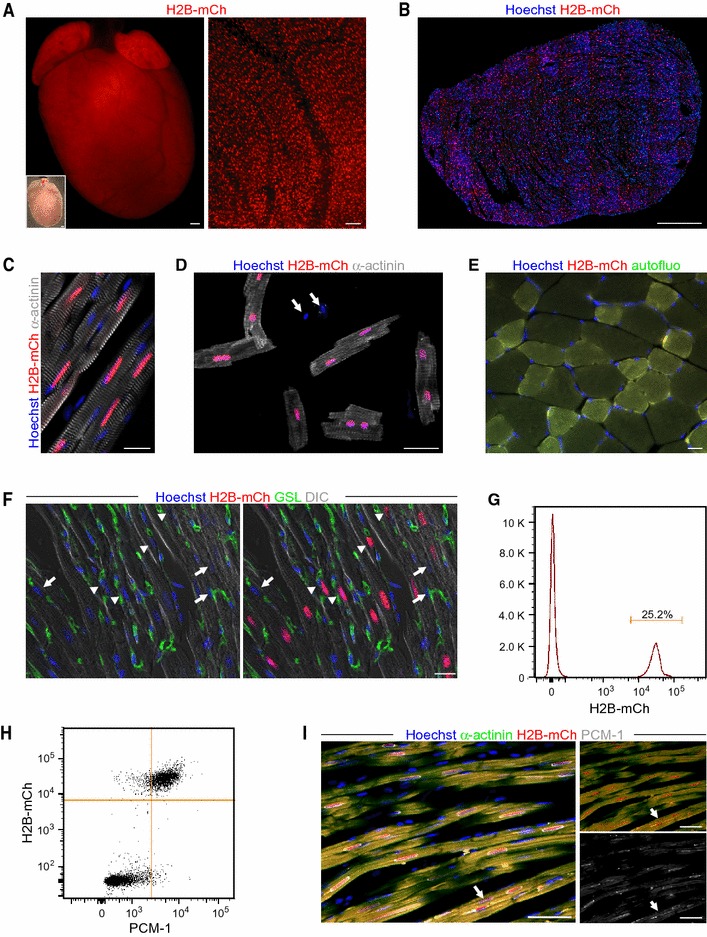
Specificity of H2B-mCh expression in CM nuclei of adult transgenic mice. **a** Macroscopic picture of an αMHC-H2B-mCh heart at adult stage. *Scale bars* 500 µm. Right picture shows a close-up of the ventricular region. *Scale bar* 100 µm. **b** Transversal section of an αMHC-H2B-mCh heart reveals expression of the fusion protein in nuclei. *Scale bar* 500 µm. **c** Section of an αMHC-H2B-mCh heart stained for α-actinin to prove expression of the fusion protein in CMs. *Scale bar* 20 µm. **d** Langendorff dissociation demonstrates expression of H2B-mCh in all CMs. *Scale bar* 50 µm. **e** H2B-mCh is not expressed in nuclei of skeletal muscle cells of H2B-mCh mice. *Scale bar* 20 µm. **f** αMHC-H2B-mCh^+^ heart section stained with GSL to identify endothelial cells. CMs were identified by cross striation in DIC. *Arrows* mark nuclei that could be misinterpreted as CM nuclei, arrowheads mark cells that would not have been identified as CM nuclei without the transgene. *Scale bar* 20 µm. **g** Representative flow cytometric analysis of the percentages of H2B-mCh^+^ and PCM-1^+^ single adult DAPI^+^ nuclei. **h** Representative flow cytometric analysis of the percentages of H2B-mCh in single adult DAPI^+^ nuclei. **i** Staining of PCM-1 and α-actinin in a cryo-slice of an αMHC-H2B-mCh heart. The *arrow* marks a CM nucleus that is not stained by PCM-1. *Scale bar* 50 µm

Macroscopically, H2B-mCh adult hearts displayed mCh fluorescence in ventricles and atria (Fig. 2a), and higher magnification showed a restriction of the H2B-mCh expression to nuclei (Fig. 2a, right picture). Cryoslices of these hearts revealed that the expression of the fusion protein was specific for all CM nuclei (Fig. 2b), which were identified by α-actinin staining (Fig. 2c).

As a definitive proof that H2B-mCh exclusively marks all CMs, we performed Langendorff dissociation of αMHC-H2B-mCh hearts (*n* = 6) to verify the specificity and the penetrance of our reporter system. All CMs with rod-shaped morphology and positive for α-actinin expressed H2B-mCh in their nuclei (Fig. 2d; *n* = 1610) proving complete penetrance, while nuclei from other cells were negative for H2B-mCh (Fig. 2d, arrows). The specificity to heart tissue was further demonstrated by analyzing sections from skeletal muscles such as M. tibialis anterior and diaphragm, which did not display any H2B-mCh expression (Fig. 2e).

We further investigated the specificity of the H2B-mCh expression pattern by staining with GSL to exclude expression in endothelial cells (Fig. 2f). There were several nuclei, which were covered by cross-striations and therefore seemingly belonged to CMs, but were clearly H2B-mCh negative (Fig. 2f, arrows) and vice versa (Fig. 2f, arrowheads). This underlines the difficulty to unequivocally identify CM nuclei on tissue sections.

Recent studies on cardiac turnover relied on the correct identification of CM nuclei by antibody staining for specific markers such as Troponin I and T [4] and PCM-1 [6], followed by flow cytometry analysis. To test, if our system can be used to identify CM nuclei out of total nuclei from hearts, we isolated cardiac nuclei from adult H2B-mCh mice and analyzed these by flow cytometry.

**Fig. 3:**
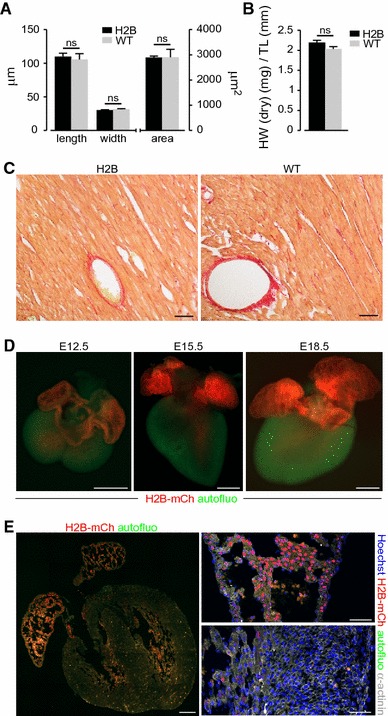
Assessment of cardiac morphology in H2B-mCh mice and H2B-mCh expression during embryonic development. **a** Exclusion of hypertrophic effects of the transgene in Langendorff dissociated adult CMs. *N* = 3 animals per group; *n* > 110 CMs per group. **b** Comparison of heart weight to tibia length between 10-week-old male αMHC-H2B-mCh transgenic and wt littermates (*n* = 3). **c** Sections of H2B-mCh and wt hearts stained with Picrosirius red for collagen. *Scale bar* 100 µm. **d** Macroscopic pictures of E12.5, E15.5, and E18.5 H2B-mCh transgenic hearts illustrate the expression of the fusion protein in atrial and some ventricular CM nuclei. *Scale bar* 500 µm. **e** Section of an E15.5 H2B-mCh heart (left picture) displays strong expression in atrial CM nuclei (right upper picture) and weak expression in some ventricular trabecular CMs (right lower picture). *Scale bars* 200 µm (overview), 50 µm (close-ups)

H2B-mCh^+^ nuclei were easily detectable by flow cytometry and contributed to 25.0 ± 4.2 % of total heart nuclei (Fig. 2g). To assess the accuracy of the aforementioned nuclear CM markers, we exemplarily stained nuclei from H2B-mCh^+^ hearts for PCM-1. Flow cytometric analysis of the stained nuclei revealed a distinct CM-population for H2B-mCh, while the PCM-1 staining displayed a broad spreading (Fig. 2h); the correlation between the two markers was 90 %, (Fig. 2h). Staining for PCM-1 on tissue sections of adult mouse hearts also revealed a good overlap with the H2B-mCh signal (Fig. 2i). However, not all of the CM nuclei could be clearly identified by PCM-1 staining (Fig. 2i, arrow). To further exclude possible side effects of the transgene on CMs, we performed morphometric analysis on Langendorff dissociated adult CMs and excluded a hypertrophic effect of transgene expression (Fig. 3a). Also the percentage of binucleated adult CMs in H2B-mCh mice [89.5 ± 2 % (*n* = 4)] matched published numbers [21, 43]. Accordingly, 10-week-old transgenic mice did not show any obvious differences in heart weight (Fig. 3b) compared to their wildtype littermates. Transgene stability was monitored for 6 generations in mice from a mixed 129S6/SvEvTac × C57BL/6Ncr × CD-1 background and for 2 generations of back-crosses into C57BL/6 background. No changes in specificity or intensity of H2B-mCh expression were noted and no detrimental effects on cardiac function and hemodynamics were observed (Table 1); in addition, no fibrosis (Fig. 3c) could be detected.

**Table 1:** Hemodynamics of H2B-mCh hearts

Parameter	WT	H2B-mCh	Significance (*p*)
Heart rate (bpm)	471.8 ± 42.6	499.7 ± 75.4	0.61
Maximum pressure (mmHg)	99.1 ± 7.9	99.6 ± 3.3	0.94
End-diastolic pressure (mmHg)	5.5 ± 5.9	4.9 ± 3.6	0.88
Stroke volume (µL)	25.5 ± 15.1	23.5 ± 7.1	0.85
Ejection fraction (%)	78.1 ± 12.4	69.4 ± 9.0	0.38
Cardiac output (µL/min)	11,787.5 ± 6295.5	11,413.9 ± 1549.7	0.93
dPdt max (mmHg/s)	9956.8 ± 1036.3	9425.9 ± 1445.6	0.63
dPdt min (mmHg/s)	−10,347.2 ± 1853.1	−10,052.9 ± 1290.4	0.83

*dPdt max* maximum rate of pressure change in the ventricle, *dPdt min* minimal rate of pressure change in the ventricle, *mmHg* millimeter of mercury (data are mean ± SEM, *n* = 3 for all)

Next, we analyzed transgene expression during embryonic development and neonatal stages. At E12.5, E15.5, and E18.5, the expression of H2B-mCh was limited mainly to the atria (Fig. 3d), probably due to the restricted activity of the αMHC promoter [22]. Although there was some expression in the trabecular zone of the left and right ventricles (Fig. 3e), the H2B-mCh signals were only detectable in a subpopulation of CMs and expression was significantly weaker compared to atrial CMs.

**Fig. 4:**
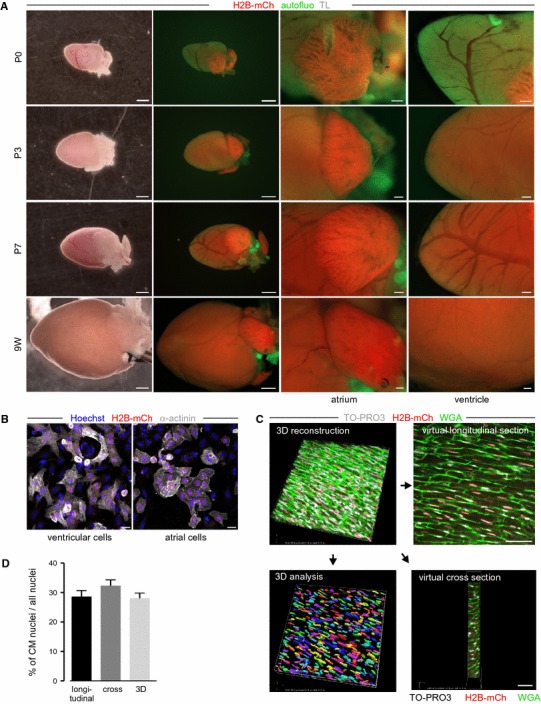
H2B-mCh transgene expression in postnatal mouse hearts. **a** Epifluorescence pictures of transgenic αMHC-H2B-mCh hearts at different postnatal days. *Scale bars* 1 mm in the overview pictures, 200 µm in close-ups of atrium and ventricle. **b** α-Actinin staining (*white*) of dissociated ventricular (left) and atrial CMs (right) of αMHC-H2B-mCh transgenic hearts proves expression of the fusion protein at P3. *Scale bars* 20 µm. **c** Illustration of three methods to quantify the portion of CM nuclei in thick slices of the heart. The 3D-analysis of H2B-mCh^+^ nuclei in correlation to all ToPro-3^+^ nuclei reflects the real fraction of CM nuclei. Virtual longitudinal sections (10 µm distance, 3–4 sections per slice) or virtual cross sections [40 µm distance, (4–5 sections per slice)] were analyzed. *Scale bars* 50 µm. Colored 3D image exemplarily depicts fragmentation of ToPro-3^+^ nuclei. **d** Quantification of the fraction of CM nuclei revealed similar results with all three methods (*n* = 3, two z-stacks per heart)

In neonatal mice, transgene expression was found to be already increased in ventricular CMs compared to late embryonic hearts (P0 in Fig. 4a) and on P3 solid expression of the H2B-mCh protein was detectable in ventricular and atrial CMs (P3 in Fig. 4a), as demonstrated by high overlap of α-actinin staining and H2B-mCh expression after dissociation (Fig. 4b).

After demonstrating the high specificity and complete penetrance of our transgene for CMs, we applied our system to several open biological questions concerning CM fractions, ploidy, and cell cycle status.

### Determination of CM nuclei fractions at postnatal stages

One focus of our study was the precise determination of the CM nuclei fraction in different areas of the heart and at different developmental time points after birth, which is of particular relevance for studying cardiac regeneration. For our analysis, we have chosen an early postnatal time point at which the heart still has proliferative capacity (P3), a later postnatal time point at which the heart is just losing its potential to regenerate (P7) and adult age (9 weeks) when the heart is terminally differentiated. Figure 4a displays epifluorescence pictures of transgenic hearts at P0, P3, P7, and at adult stage [9 weeks (9 W)]. While the expression level of H2B-mCh increased in the ventricle during postnatal development, it did not change in atria (Fig. 4a, close-ups).

For quantification of CM nuclei fractions, we analyzed heart sections for H2B-mCh^+^ nuclei and total cardiac nuclei. We assumed that due to the occurrence of binucleated CMs, there would be a difference between quantification of CM nuclei based on either transverse or cross sections. In a cross section, only one of the two nuclei of a binucleated CM can be visible, while in longitudinal sections the chance to detect both nuclei is limited by section thickness. Therefore, both methods could be error-prone and either underestimate or overestimate the number of CMs. To determine the difference of counting transverse or cross sections on actual CM numbers, we first quantified the fraction of CM nuclei in volume 3D-reconstitutions of Z-stacks of thick (50 µm) slices from adult αMHC-H2B-mCh hearts without taking into account the degree of binucleation. Nuclei were stained with TO-PRO3 iodide, cell borders were visualized by WGA staining and CM nuclei were identified by nuclear H2B-mCh fluorescence (Fig. 4c). The effect of quantification of longitudinal or cross cardiac sections was determined by generating virtual sections from the 3D volume view (Fig. 4c). Interestingly, both methods of quantification gave very similar results compared to the fraction of CM nuclei obtained by analyzing the 3D reconstitution (Fig. 4d).

**Fig. 5:**
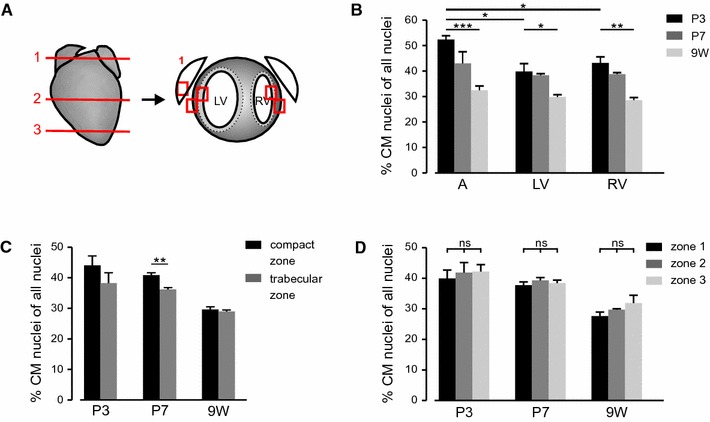
Determination of the percentage of CM nuclei at different developmental stages. **a** Scheme illustrates the strategy used for analyzing the CM nuclei proportion in different regions of the heart. **b** Quantification of the percentage of CM nuclei at different developmental stages. *A* atrium, *LV* left ventricle, *RV* right ventricle, (*n* = 3). **c** Compact zone compared to trabecular region. **d** Fraction of CMs in different zones of the heart as described in (**a**)

To assess potential regional differences in the fraction of CM nuclei at each time point, which could help identifying the most appropriate region(s) for proliferation inducing therapeutic approaches, different zones along the longitudinal axis of the heart (zone 1–3) were analyzed as depicted in Fig. 5a; in these areas we further distinguished between atria, left (LV) and right ventricle (RV) as well as between the compact and trabecular zone. The CM nuclei fractions were quantified by counting H2B-mCh^+^ nuclei and total number of nuclei. Quantification revealed a significant decrease in the fraction of CM nuclei from P3 to 9 W in all analyzed areas (Fig. 5b). At P3 a significantly higher fraction of CM nuclei was observed in atria compared to LV, while the CM nuclei fractions did not differ significantly between the ventricles. At P7 the difference between CM nuclei fractions in atria and ventricles was evened and in 9 W old hearts the fraction of CM nuclei was ~30 % in atria as well as in both ventricles (Fig. 5b). These values were verified by flow cytometric analysis of isolated nuclei from hearts of 10-week-old transgenic animals, in which 25.0 ± 4.2 % of nuclei turned out to be H2B-mCh^+^ (Fig. 2g). Regarding regional differences within the ventricles, a significantly smaller portion of CM nuclei was found in the trabecular zone compared to the compact zone on P3 and P7 (Fig. 5c). However, after compaction in adult hearts, the portion of CM nuclei was comparable in both zones (Fig. 5c). Along the apical- to basal heart axis, the CM nuclei fraction in the ventricles did not differ significantly (Fig. 5d). However, there was a slight tendency toward higher fractions of CM nuclei in apical direction. For accurate determination of CM number, the nuclearity of the CMs had to be taken into account, because most of the CMs in mice are binuclear [37].

### Determination of CM nuclearity and CM fractions at postnatal stages

**Fig. 6:**
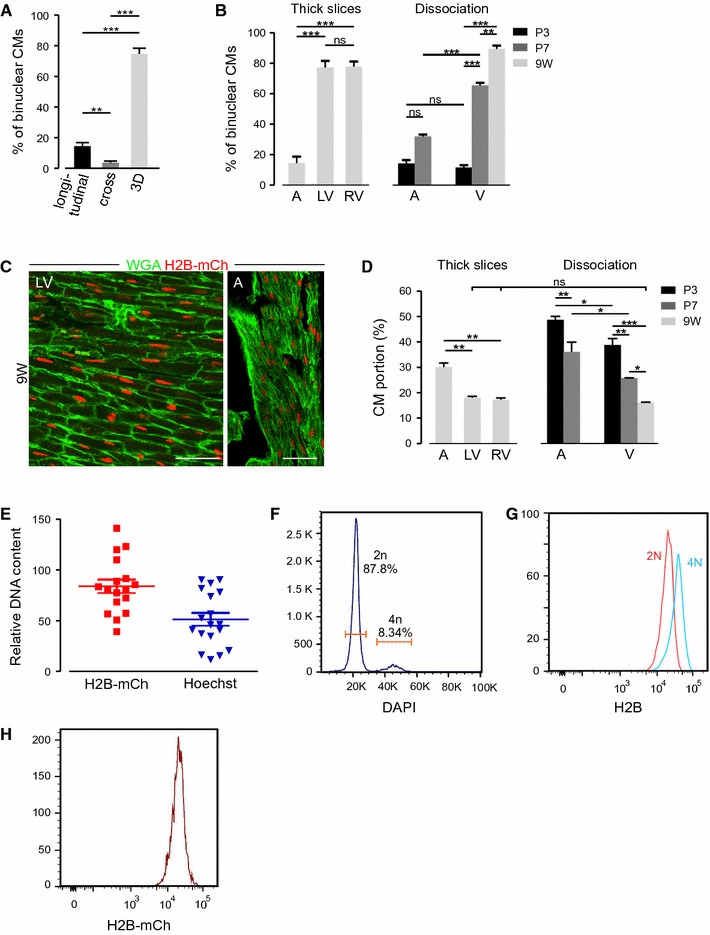
Determination of nuclearity and DNA content in H2B-mCh CMs. **a** Portion of CMs with two nuclei in one section (*n* = 3, 2 z-stacks per heart) determined in thick slice 3D reconstruction and virtual longitudinal and cross sections. **b** Quantification of the percentage of binuclear CMs in *LV* left ventricle and *RV* right ventricle at P3 (*n* ≥ 3), P7 (*n* ≥ 3), 9 W (*n* = 3), and in *A* atrium at 9 W (*n* = 2). Quantification was performed in z-stacks of thick slices for 9 W and cell dissociation for P3, P7, and 9 W. **c** Single z-layers of thick heart slices show differences in the tissue morphology of the *LV* left ventricle and *A* atrium at adult stage. Membranes are stained with Wheat germ agglutinin (*green*). *Scale bar* 50 µm. **d** Calculation of the portion of CMs taking into account the numbers of Fig. 5b and the grade of binuclearity from thick slices and cell dissociation. **e** Relative DNA content of Langendorff isolated CMs as assessed by H2B-mCh intensity or Hoechst intensity, respectively. **f** Representative flow cytometric analysis of the ploidy of H2B-mCh single adult DAPI positive nuclei. **g** Representative flow cytometric analysis of the fluorescence intensity of mCh from diploid and tetraploid CM nuclei. **h** Representative flow cytometric analysis of the fluorescence intensity of mCh^+^ nuclei

Shortly after birth CMs, undergo cell cycle variations resulting in binucleated and polyploid cells. These CMs are thought to be terminally differentiated [35], while there is evidence that mononucleated, diploid CMs have the potential to divide after stimulation of neuregulin/ErbB4-signaling [7] or after experimental myocardial lesion [7, 33]. To determine the actual number of CMs from the fraction of CM nuclei and due to the potential relevance of mononuclear CMs for regenerative approaches in the heart, we quantified the fraction of bi- and mononucleated CMs at different time points and in different regions of the heart. However, the degree of binucleated CMs could not be determined accurately with either cross sections or longitudinal sections compared to 3D reconstitution (Fig. 6a). This is due to general heart geometry and the orientation of CMs in heart tissue, in which muscle fibers are curved and CMs are oriented at different angles. Furthermore, in an optimal standard cross section (5–10 µm) binucleated cells cannot be detected, because usually only one nucleus is sectioned at a time. In longitudinal sections, the thickness of a standard section (5–10 µm) limits the detection of both nuclei in binucleated CMs, if the CMs are not oriented in parallel to the sectioning plane or nuclei of one CM are not completely located in the same plane.

To accurately determine the ratio of mononucleated to bi- or multinucleated CMs, we followed two different approaches. The number of nuclei per CM was counted in enzymatically dissociated transgenic hearts at P3, P7, and in Langendorff-dissociated hearts at 9 W. The quantification of isolated CMs revealed a strongly increased binucleation index in ventricular CMs from 11.6 ± 1.6 % at P3, to 65.5 ± 1.7 % at P7, and to 89.5 ± 2.0 % at 9 W (Fig. 6b). In atrial CMs, we determined only a slight increase in binuclearity from P3 (14.2 ± 2.2 %) to P7 (32.0 ± 1.2 %). The fraction of multinucleated CMs exceeding binucleation was <1 % in all cases. In parallel, confocal images of thick slices (50 µm) of transgenic hearts at 9 W were analyzed as Z-stacks to exclude a potential selective loss of mono- or binucleated CMs during the Langendorff isolation procedure. The slices were stained with WGA to visualize cell borders (Fig. 6c). Only CMs that lay completely within the z-stack (WGA staining visible in every dimension) were analyzed.

The quantifications of Z-stacks at adult stage allowed a separate analysis of the binucleation index in cells from the left (77.2 ± 4.4 %; *n* = 5) and right ventricle (77.8 ± 3.4 %; *n* = 5) and the atrium (14.4 ± 4.3 %; *n* = 4) (Fig. 6b), thereby supporting the results obtained by the analysis of the Langendorff dissociated CMs. The fraction of mononucleated cells in the ventricles, as determined by this method, was significantly higher (*p* = 0.034) than with enzymatic dissociation.

By taking into account the portion of binucleated CMs, as assessed by Z-stack analysis, enzymatic cell separation and Langendorff dissociation, we were able to calculate the portions of CM cells in atria and ventricles at the selected time points (Fig. 6d). Due to the low degree of binucleation in atria, the fraction of CMs in this compartment was significantly higher at all analyzed stages (P3: 48.7 ± 1.4 %; P7: 36.1 ± 3.9 %; 9 W: 30.1 ± 1.6 %) compared to ventricles at P3 (39.2 ± 2.6 %), P7 (25.8 ± 0.1 %), 9 W (16.1 ± 0.2 % dissociation) and LV (18.1 ± 0.6 % thick slices) and RV (17.3 ± 0.6 % thick slices), respectively.

We also explored, whether H2B-mCh expression correlates to DNA content and could therefore be used as a direct readout for endoreduplication. However, neither Hoechst staining of thick sections of the LV (Fig. 6e) nor isolation of CM nuclei followed by nuclear staining and flow cytometry (Fig. 6f–h) yielded a direct correlation between H2B-mCh signal intensity and DNA content.

### Easy identification of the border zone after cryoinjury of the heart

**Fig. 7:**
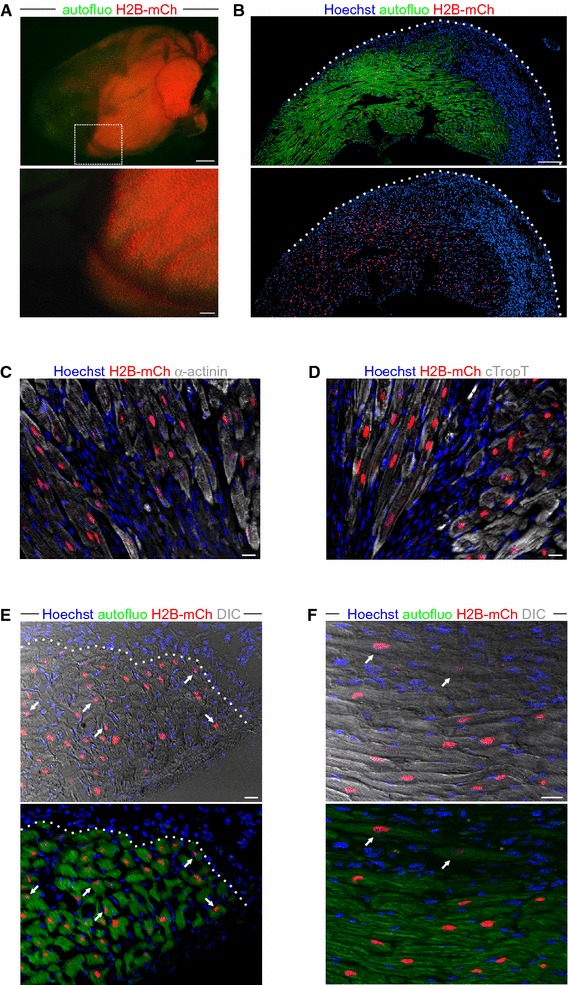
Visualization of CM nuclei after cardiac infarction. **a** Macroscopic pictures of an αMHC-H2B-mCh heart 10 days after cryoinjury. Close-up depicts border zone region. *Scale bars* overview: 1 mm, close-up: 200 µm. **b** Section of a cryo-infarcted αMHC-H2B-mCh heart. H2B-mCh signals are only abundant in intact CMs and are lost in the infarcted region. *Scale bar* 200 µm. (**c**, **d**) αMHC-H2B-mCh^+^ heart section stained with either α-actinin (**c**) or cardiac Troponin T (**d**) to identify CMs. Good co-localization between the cardiac markers and H2B-mCh in the border zone. *Scale bars* 20 µm. **e** H2B-mCh expression facilitates the identification of CM nuclei in cross sections of the border zone. *Arrows* depict CM nuclei, which are not centered in the cell. *Scale bar* 20 µm. **f** H2B-mCh expression facilitates the identification of individual surviving CMs (*arrows*) within the lesioned area. *Scale bar* 20 µm

After experimental myocardial infarction, it is challenging to clearly identify and quantify infarct size and surviving CMs in the border zone and infarcted area. This is especially true for islands of surviving CMs within the infarct. Additionally, staining for antigens in the infarct zone is difficult due to high background issues. We thought that these difficulties could be addressed by the prominent in vivo labeling of CM nuclei and therefore analyzed cardiac sections 10 days after the injury (*n* = 2). The lesioned area and the native myocardial tissue were easy to discriminate macroscopically by H2B-mCh signals (Fig. 7a). Staining of sections from lesioned transgenic hearts revealed a good demarcation of the infarct area and the viable myocardium with H2B-mCh^+^ nuclei (Fig. 7b). Specificity of the H2B-mCh signal was corroborated by staining for α-actinin (Fig. 7c) and cardiac troponin T (Fig. 7d).

The expression of the fusion protein further facilitates the identification of nuclei from border zone CMs. Especially in cross sections, the reliable identification of CM nuclei is limited without the use of a specific nuclear marker as the CM nuclei are not always centered within the cells (Fig. 7e, arrows). Similarly, individual CMs or small islands of surviving CMs, which are hard to detect within the lesioned area, are easily detectable by their H2B-mCh nuclear signal (Fig. 7f, arrows).

In summary, the αMHC-H2B-mCh mouse line facilitates the reliable identification of CM nuclei after myocardial infarction and eases the estimation of the lesion size.

### An in vitro assay for screening for proliferation-inducing substances in CMs

**Fig. 8:**
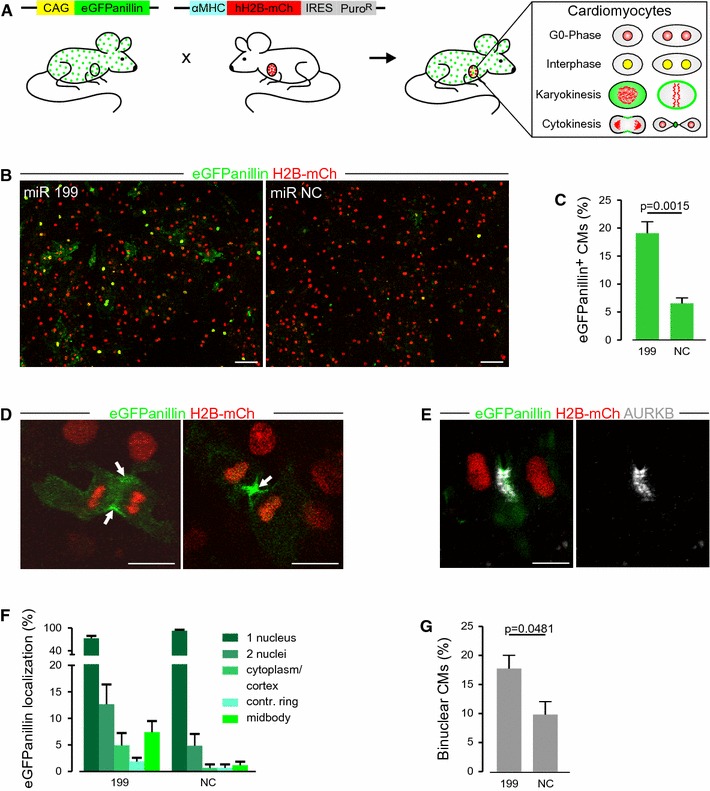
Assay for screening the effects of cell cycle modifying substances on CMs. **a** Scheme depicts the cross-breeding of αMHC-H2B-mCh mice with the CAG-eGFP-anillin proliferation indicator mouse. Depending on the cell cycle status, CMs (H2B-mCh^+^ nuclei) express eGFP-anillin in different subcellular localizations. **b** Fluorescence pictures of dissociated αMHC-H2B-mCh/CAG-eGFP-anillin double transgenic hearts (P2), transfected with the cell cycle-modifying miR-199 and a miR-NC. *Scale bars* 100 µm. **c** Quantification of eGFP-anillin expression in miR-treated CMs 72 h after transfection (*n* ≥ 3). **d** Examples of CMs with cytokinesis-indicating eGFP-anillin localizations (arrows). *Scale bars* 100 µm. **e** Staining of eGFP-anillin/H2B-mCh CMs with the proliferation marker *AURKB* Aurora B kinase. Note the overlap between the M-phase specific localization of eGFP-anillin (*green*) contractile ring and Aurora B kinase (*white*). *Scale bar* 10 µm. **f** Analysis of different eGFP-anillin localizations in CMs after miR-treatment (*n* ≥ 3). **g** Portion of binuclear CMs 72 h after miR-transfection (*n* ≥ 3)

A potentially very interesting strategy to regenerate lost heart muscle is based on re-induction of proliferation in pre-existing CMs. For this purpose preferentially neonatal stages are used, as at this stage some proliferative activity is still present in CMs and these cells appear to be more amenable to cell cycle-modulating interventions. To establish an in vitro and in vivo screening system for such factors and substances, we crossed the αMHC-H2B-mCh mice with mice expressing the CAG-eGFP-anillin transgene [15], which visualizes cell cycle activity with high resolution of M-phase. In this double transgenic mouse line, CMs can be identified by nuclear expression of H2B-mCh and their cell cycle status by localization of the eGFP-anillin fusion protein (Fig. 8a). As proof of concept, we used postnatal CMs derived from double transgenic mice to test the influence of substances on cell cycle activity and proliferative behavior in vitro. We chose P2 mice for the experiment, as this age demarcates the time frame in which CMs are still proliferative, but will soon undergo the transition to binucleation, starting around P4. As the experiment lasts 3 days, which corresponds to P3–P5, both regular proliferation as well as cell cycle variations will take place, thereby providing a challenge for the detection system to correctly distinguish between the two states. Ventricles from P2 mice were dissociated and the cells were transfected with either microRNA 199, (miR-199) which was reported to strongly enhance the rate of proliferation in postnatal CMs [10] or scramble miRs as a negative control. By counting eGFP-anillin expressing CMs, which could be identified according to their H2B-mCh fluorescence, increased cell cycle activity after treatment with miR-199 compared to the scramble miR control could be directly detected without further stainings (Fig. 8b). While in transfections with scramble miRs, a basal cell cycle activity (6.6 %) could be determined, this was significantly (*p* = 0.0015) enhanced (19.1 %) after transfection with miR-199 (Fig. 8c). Importantly, the fraction of non-nuclear localizations of the eGFP-anillin signal, such as contractile rings and midbodies (Fig. 8d), which are indicative for cell division [15] and were verified by staining for Aurora B kinase (Fig. 8e), was increased after miR-199 treatment compared to the control (Fig. 8f). Further we noticed an increase in the fraction of binucleated CMs in miR-199-treated CMs (Fig. 8g). As the strength of our system is live-tracking of cell cycle progression, we performed video microscopy, which revealed both CMs’ divisions (Suppl. Video 2) as well as binucleation (Suppl. Videos 3 + 4) taking place. Binucleation was determined as 17.7 % of total CMs for miR-199 treatment and only 9.8 % for controls. This experiment demonstrates that the αMHC-H2B-mCh/CAG-eGFP-anillin system not only enables the identification of proliferation, but also of cell cycle variations such as binucleation. Therefore, it is ideally suited for screening of cell cycle-modifying substances in CMs.

## Discussion

We have generated a novel transgenic model for the direct visualization of CM nuclei in vitro and in vivo. This system is based on the expression of a fusion protein consisting of the human histone 2B and the red fluorescent protein mCh under transcriptional control of the CM specific Myh6 promoter, resulting in a nuclear signal. We showed the specific labeling of CM nuclei throughout our in vitro and in vivo experiments.

As the mammalian heart consists of mono- and binucleated CMs, it was of great interest to determine the fraction and nuclearity of CMs in different regions of the mouse heart. The contribution of CMs to total number of heart cells in adult humans and rodents has been estimated to be 15–20 % [37, 42]. However, for mice, even substantially higher estimates have been reported, assigning an exceptional role for this species [2]. We can clearly exclude this and confirm earlier estimates of <15 % CMs, which correspond to <30 % CM nuclei [37, 43]. While previous quantifications were based on cell suspensions of dissociated hearts, our data are based on (1) flow cytometry of isolated nuclei, (2) quantification on tissue sections, (3) Langendorff isolation, as well as (4) 3D-reconstruction and (5) Z-stack analysis of cardiac slices. Cell isolation procedures in general cannot guarantee uniform extraction of all cell types. Cells that are more sensitive to enzymatic treatment are prone to be damaged, while cells surrounded by connective tissue are less likely to be dissolved from the tissue. By using Z-stacks of cardiac sections for quantitation, we found a significantly lower fraction of binuclear CMs as with enzymatic dissociation. A recent study in which histological sections were used also reported higher numbers of mononuclear CMs (24 % [33]) that match our observations of ~25 %. In atria of adult mice, we found an even higher fraction of mononucleated cells of ~86 %. The functional difference between mono- and binucleated CMs is unknown. However, there is evidence that mononucleated CMs are prone to enter the cell cycle after stimulation with neuregulin [7] or after cardiac lesion [33]. Interestingly, atrial mononuclear CMs display cell cycle activity after experimental lesion in the left ventricle in rats, which was attributed to either cell division [32] or increased binucleation [28].

Additionally, we also addressed the issue of CM fractions in the context of cardiac growth and spatial distribution in the heart, which is of relevance for studying cardiac regeneration as it has been shown that neonate mammalian hearts have a remarkable regenerative capacity [30]. We found a significantly higher proportion of CMs at P3 compared to P7 and adult mice which is most likely due to the extensive proliferation of fibroblasts taking place from P1 to P10 [37]. The spatial distribution of CMs revealed only a difference in atria of P3, P7, and adult mice, in which the fraction of CMs was significantly higher compared to the ventricles. This is due to the significantly lower fraction of binucleated cells in atria of mouse, which was ~14 % in adults.

A promising strategy for regeneration of lost CMs is the re-induction of CM division by application of proliferation-inducing substances. However to identify such factors and to verify them in vivo, CM nuclei and cell division need to be unequivocally identified.

The advantages of our CM proliferation assessment assay, which is based on H2B-mCh/eGFP-anillin compound transgenic mice, are the immediate CM nuclei identification and quantification of cell cycle activity due to the fluorescent labels and in particular the possibility to live-monitor cell cycle progression of CMs. In contrast, most commonly used assays use BrdU and stainings with combinations of Ki-67, pHH3, PCNA, and AuroraB-kinase [7, 9, 10, 41] which require fixation and prevent the direct follow-up of cell fate. As a proof of concept, we were able to verify the proliferation-inducing effect of miR-199, as previously published [10]. Due to the high resolution of M-phase, the eGFP-anillin assay also allows us to discriminate between cell division and cell cycle variations such as endoreduplication and acytokinetic mitosis leading to binucleation [15]. Surprisingly, treatment with miR-199 led to an increase of binucleated CMs, indicating induction of cell cycle variations. In summary, the H2B-mCh/eGFP-anillin assay will provide crucial information about the true nature of cell cycle activity in the future.

Currently, identification of CMs is mostly based on staining of the contractile apparatus (cTropI, cTropT or α-actinin) or on the use of transgenic mouse models such as αMHC-mCh mice [19] or αMHC-Cre inducible mice [34] which provide CM-specific cytoplasmic expression of fluorescence reporters [18]. As a caveat, these techniques do not allow a direct correlation of nuclei to the marked CMs. False positive identification of a non-CM nucleus as cell cycle active CM nucleus may strongly alter a conclusion concerning the regeneration potential of adult CMs as the numbers of CMs with cell cycle activity are described to be very low (1 in 180,000) [36]. Indeed, a lack of proper identification of CM nuclei seems to be one of the major reasons for the high divergences in literature concerning the annual turnover rate of adult mouse CMs, ranging from 0.76 % [33] up to 80 % [17].

Currently only one further transgenic mouse model, MHC-nLAC, has been described which enables a specific identification of CM nuclei by staining for β-galactosidase activity [38]. As a shortcoming, the nuclear signal is replaced by a diffuse cytoplasmic signal in CMs as soon as the nuclear envelope dissolves. This complicates quantifications of CM nuclei if cells enter M-Phase. The H2B-mCh mice described here have two advantages over the existing mouse lines, which use NLS or cytoplasmic expression of a CM specific marker: first, compared to lacZ as a marker [38] no additional staining step is needed, which is particulary important for live-imaging applications and second, due to the fusion to histone H2B, mCh stays connected to the chromosomes during all phases of the cell cycle, a prerequisite for automatic cell tracking in a screening assay [27]. Furthermore, our mouse model can also be used to directly and specifically isolate CM nuclei for the analysis of epigenetic chromatin modifications, an approach recently established in CMs [13]. Taken together, our transgenic system provides a new technology for the identification of CM nuclei without any further treatment and for the isolation of CM nuclei for downstream applications. In combination with the eGFP-anillin system, it is a novel tool to specifically resolve controversial questions in the cardiac field, such as the ongoing debate, if adult CMs have the potential to divide. This mouse line will be useful for assessing proliferation in models of cardiac regeneration, for the differentiation between atrial and ventricular CMs during embryonic development, identification of CMs in screening assays for proliferation-inducing substances, transplantation experiments, and for the analysis of plasticity of adult CMs in future studies.

## Electronic supplementary material

Supplementary material 1 (AVI 8393 kb)

Supplementary material 2 (AVI 1906 kb)

Supplementary material 3 (AVI 11048 kb)

Supplementary material 4 (AVI 11207 kb)

Supplementary material 5 (DOC 28 kb)
