# Supplementary material for: Transgenic systems for unequivocal identification of cardiac myocyte nuclei and analysis of cardiomyocyte cell cycle status
Source: Basic Res Cardiol. 2015 Apr 30;110(3):33. doi: 10.1007/s00395-015-0489-2 (PMC4414935; doi:10.1007/s00395-015-0489-2)
Supplement: Supplementary file 5 — Supplementary material 5 (DOC 28 kb) [file 395_2015_489_MOESM5_ESM.doc]

**Supplemental Material**

**Supplemental Videos**

**Video 1:**

Video of spontaneously beating αMHC-H2B-mCh transgenic EBs on d12 of differentiation. Expression of the fusion protein is restricted to the beating clusters indicating expression in CMs. Recording and display frame rate is 15 frames s^−1^.

**Video 2:**

Time lapse imaging of a transgenic αMHC-H2B-mCh/CAG-eGFP-anillin transgenic postnatal CM (isolated and transfected with miR 199 on P2, imaged 2 days later) that undergoes cell-division. The midbody appears between the daughter nuclei, and persists there after abscision. The daughter nuclei depart from each other. Pictures were taken every 5 min. and frame rate is 5 s^−1^.

**Video 3:**

Time lapse imaging of a transgenic αMHC-H2B-mCh/CAG-eGFP-anillin transgenic postnatal CM (isolated and transfected with miR 199 on P2, imaged 2 days later) that becomes binucleated. The constriction of the contractile ring leads to the formation of a midbody. However, the midbody is directly fragmented. Abscission does not take place and the daughter nuclei stay close to each other. Pictures were taken every 5 min. and frame rate is 3.5 s^−1^.

**Video 4:**

Time lapse imaging of a transgenic αMHC-H2B-mCh/CAG-eGFP-anillin transgenic postnatal CM (isolated and transfected with miR 199 on P2, imaged 2 days later) that becomes binucleated. The constriction of the contractile ring leads to the formation of a midbody. However, the midbody directly translocates to a non-central position at one side of the former contractile ring. Abscission does not take place and the daughter nuclei stay close to each other. Pictures were taken every 5 min. and frame rate is 3.5 s^−1^.
